# RETRACTION: Effects of Evidence‐Based Nursing on Surgical Site Wound Infection in Patients Undergoing Acute Appendicitis Surgery: A Meta‐Analysis

**DOI:** 10.1111/iwj.70286

**Published:** 2025-03-03

## Abstract

**RETRACTION:** LiuF. , ZhouJ. , and WuX.‐L. , “Effects of Evidence‐Based Nursing on Surgical Site Wound Infection in Patients Undergoing Acute Appendicitis Surgery: A Meta‐Analysis,” International Wound Journal 21, no. 3 (2024): e14539, 10.1111/iwj.14539.38506317 PMC10952117

The above article, published online on 20 March 2024, in Wiley Online Library (http://onlinelibrary.wiley.com/), has been retracted by agreement between the journal Editor in Chief, Professor Keith Harding; and John Wiley & Sons Ltd. Following an investigation by the publisher, all parties have concluded that this article was accepted solely on the basis of a compromised peer review process. The editors have therefore decided to retract the article. The authors did not respond to our notice regarding the retraction.

**RETRACTION:** F. Liu , J. Zhou , and X.‐L. Wu , “Effects of Evidence‐Based Nursing on Surgical Site Wound Infection in Patients Undergoing Acute Appendicitis Surgery: A Meta‐Analysis,” International Wound Journal 21, no. 3 (2024): e14539, 10.1111/iwj.14539.38506317 PMC10952117

The above article, published online on 20 March 2024, in Wiley Online Library (http://onlinelibrary.wiley.com/), has been retracted by agreement between the journal Editor in Chief, Professor Keith Harding; and John Wiley & Sons Ltd. Following an investigation by the publisher, all parties have concluded that this article was accepted solely on the basis of a compromised peer review process. The editors have therefore decided to retract the article. The authors did not respond to our notice regarding the retraction.

